# Case report: A case of unilateral combined central retinal vein occlusion, incomplete central retinal artery occlusion, and papillitis following a third dose of COVID-19 vaccination

**DOI:** 10.3389/fopht.2024.1352962

**Published:** 2024-02-23

**Authors:** Ami Furukawa, Yukihiko Suzuki, Narumi Nozuki, Naruki Kurosaka, Satomi Kogawa, Shinya Hara, Shinji Ueno

**Affiliations:** ^1^ Department of Ophthalmology, Hirosaki University Graduate School of Medicine, Hirosaki, Japan; ^2^ Hara Eye Clinic, Goshogawara, Japan

**Keywords:** COVID-19 vaccination, central retinal vein occlusion, incomplete central retinal artery occlusion, papillitis, macular edema

## Abstract

**Purpose:**

The aim of this study was to present a case of severe visual loss due to retinal arteriovenous occlusion and papillitis in one eye following vaccination against coronavirus disease (COVID-19).

**Methods:**

A 45-year-old man undergoing treatment for hypertension had severely reduced visual acuity in the right eye 1 day after receiving a third dose of a COVID-19 vaccine manufactured by Moderna. Clinical examination showed that the best-corrected visual acuity in the right eye was counting fingers. Other findings included circumferential retinal hemorrhage, perimacular ischemic color, severe macular edema, and severe optic disc swelling, indicating the presence of central retinal vein occlusion, incomplete central retinal artery occlusion, and papillitis. Based on the possibility of post-vaccination inflammation and/or abnormal immune response, three courses of steroid pulse therapy were administered, and the visual acuity slightly improved to 20/1,000.

**Results:**

Three months after the onset of symptoms, macular edema disappeared; conversely, retinal thinning of the macula and extensive non-perfusion areas mainly on the nasal side were noted.

**Conclusion:**

The findings in this case suggest that inflammation and abnormal immune response after receiving a COVID-19 vaccination may lead to combined retinal arteriovenous occlusion and papillitis.

## Introduction

A variety of vaccines have been produced and used by millions of individuals since the 2019 global outbreak of the coronavirus disease (COVID-19). Nevertheless, several reports have been presented regarding what may be considered eye-related side effects (1). These side effects include conditions such as optic neuritis, optic perineuritis, arteritic anterior ischemic optic neuropathy, non-arteritic anterior ischemic optic neuropathy, acute zonal occult outer retinopathy (AZOOR), acute macular neuroretinopathy (AMN) (2, 3), paracentral acute middle maculopathy (PAMM) (3), branch retinal artery occlusion (BRAO) (2), central retinal vein occlusion (CRVO) (4, 5), branch retinal vein occlusion (BRVO), combined arterial and venous occlusion (2), and combined central retinal artery and vein occlusion with ischemic optic neuropathy (6).

## Case report

A 45-year-old man with a history of hypertension being treated medically, received a third vaccination for COVID-19 in July 2022. Each was with the mRNA COVID-19 vaccine manufactured by Moderna. That day, the patient had no fever or other systemic symptoms noted, although the next morning, he noticed a severe visual impairment in his right eye and came to our department soon thereafter.

**Figure 1:**
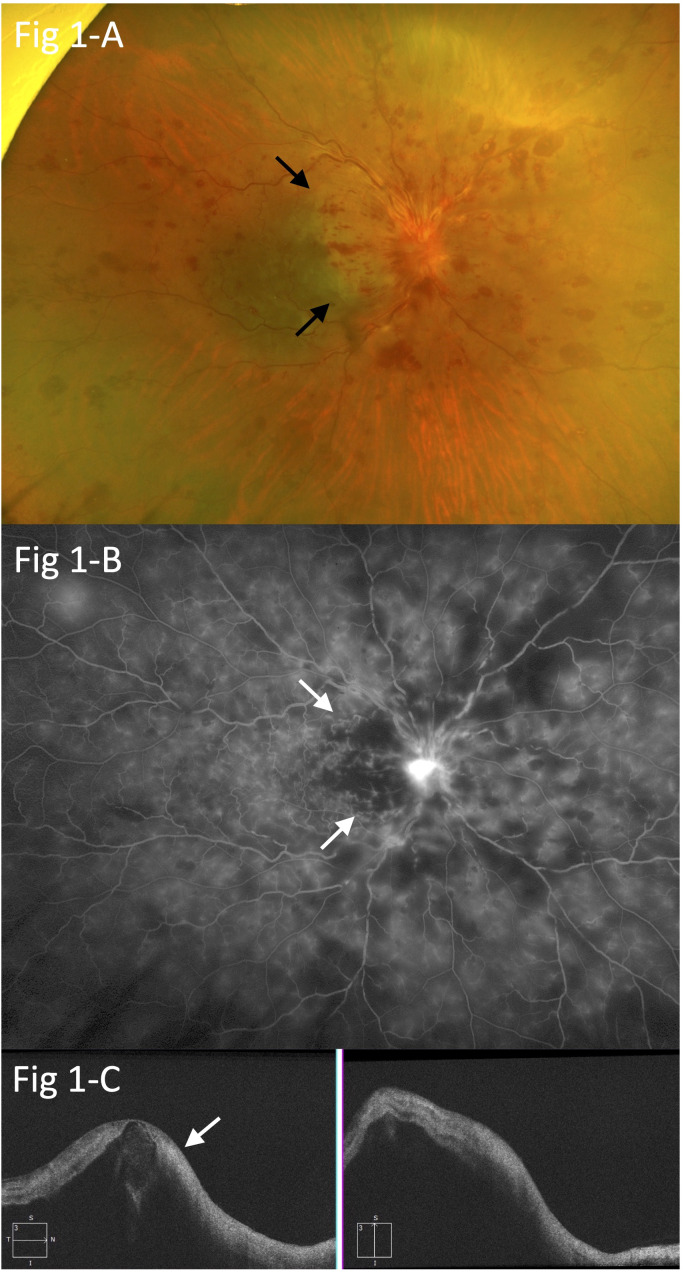
**(A)** Circumferential retinal hemorrhage, perimacular ischemic color (arrows), severe macular edema, and severe optic disc edema, suggesting central retinal vein occlusion (CRVO), branch retinal artery occlusion (BRAO), and papillitis. **(B)** Fluorescein angiography (FA) showing prolonged arm-to-retina circulation and intra-retinal transitional times, perimacular filling defects (arrows), and blockage of fluorescein due to retinal hemorrhage in the right eye. **(C)** Optical coherence tomography (OCT) findings indicating severe macular edema and hyperintensity in the inner retina (arrow).

At the initial visit, visual acuity was limited to counting fingers in the right eye and 30/20 in the left eye. A relative afferent pupillary defect was also noted in the right eye, and additional examinations showed circumferential retinal hemorrhage, perimacular ischemic color, severe macular edema, and severe optic disc edema, indicating the presence of central retinal vein occlusion (CRVO), incomplete central retinal artery occlusion (incomplete CRAO), and papillitis (**Figure 1A**). Fluorescein angiography (FA) indicated prolonged arm-to-retina circulation and intraretinal transitional times, perimacular filling defects, and blockage of fluorescein due to the retinal hemorrhage (**Figure 1B**). Optical coherence tomography (OCT) revealed severe macular edema and hyperintensity of the inner retina (**Figure 1C**). In addition, the retina of the left eye showed increased arteriovenous crossings, suggesting the presence of retinal arteriolosclerosis, although no retinal hemorrhage was observed.

In addition to the simultaneous onset of CRVO and incomplete CRAO in the right eye, papillitis was suspected. Contrast MRI showed no contrast enhancement of the optic nerve itself, suggesting inflammation confined to the optic nerve disc rather than inflammation of the optic nerve itself, as seen in idiopathic optic neuritis or antibody-related optic neuropathy.

Since the patient was thought to be affected by CRVO and incomplete CRAO, both of which can cause macular edema, it was difficult to choose an anti-VEGF drug treatment that could quickly reduce macular edema caused by CRVO. Furthermore, papillitis was also likely a complicating factor, and the resulting inflammation may have caused circulatory disturbance of retinal blood vessels; thus, steroid pulse therapy was chosen to reduce inflammation as much as possible, with a full explanation given to the patient. Although steroid pulse therapy is often completed after one or two courses, it was decided that three courses be performed over a 3-week period because the patient had severely impaired vision and was a relatively young man with no underlying disease other than hypertension. In the present case, one course of steroid pulse therapy consisted of intravenous methylprednisolone 1,000 mg/day for 3 days, followed by rest for the remaining 4 days.

**Figure 2:**
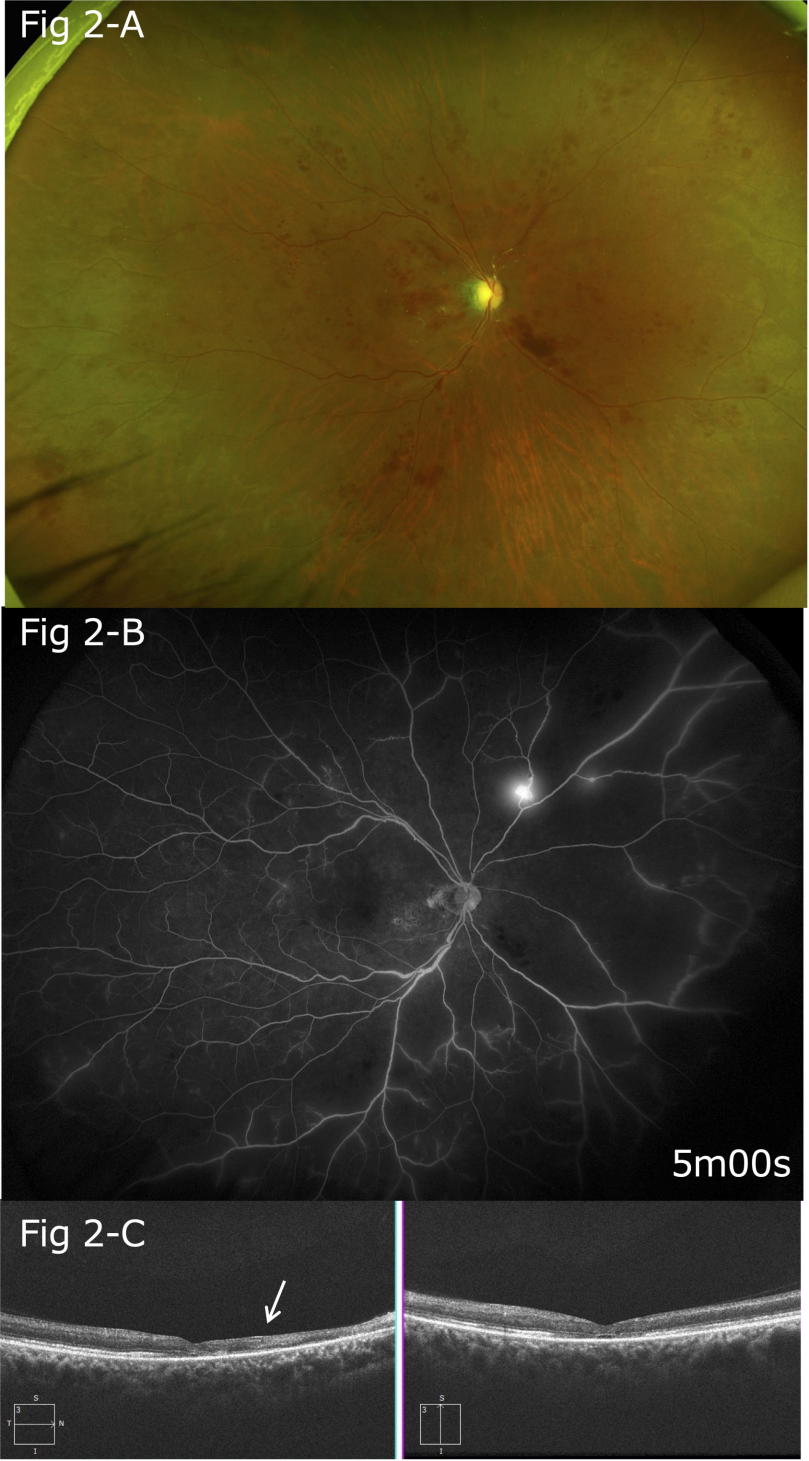
**(A)** Five months after the onset, gradual improvements in optic disc edema, macular edema, and retinal hemorrhage were noted. **(B)** Follow-up FA findings showing extensive non-perfusion areas, mainly on the nasal side, and leakage of fluorescein from the neovascular vessels on the superior nasal side. **(C)** After five months, thinning of the retina was particularly apparent between the optic disc and macula (arrow).

Platelet aggregation test findings showed that the maximum aggregation rate with the use of aggregation-inducing substances increased to 80% with 1 μM of adenosine diphosphate (ADP) and 84% with 0.5 μg/mL of collagen. Blood examination results showed increased platelet aggregation; thus, oral aspirin 100 mg/day was also started. Findings of optic disc edema, macular edema, and retinal hemorrhage demonstrated gradual improvements, and visual acuity in the right eye increased to 20/1,000 (**Figures 2A, C**).

Three months after the onset of the conditions, extensive non-perfusion areas were observed in the FA images, mainly on the nasal side. Pan-retinal photocoagulation was performed 2 months later because FA revealed retinal neovasculization. FA findings obtained at that time also indicated extensive non-perfusion areas, mainly on the nasal side, as well as leakage of fluorescein from the neovascular vessels on the superior nasal side (**Figure 2B**, arrowhead).

## Discussion

Retinal complications in COVID-19 cases have been reported to include microinfarction and retinal hemorrhage (7), as well as AMN and PAMM. Furthermore, the same study noted the occurrence of retinal abnormalities (e.g., cotton wool spots and retinal hemorrhage) in 12% of patients hospitalized for severe systemic conditions related to COVID-19.

Although several cases of possible adverse reactions to vaccines have been noted in recent years, there have been few reports of retinal arteriovenous occlusive disease, such as those seen in the present case. The patient in this case was only 45 years old, although he had a history of hypertension. The findings of increased arteriovenous crossing of the retina in the left eye suggested the presence of retinal arteriolosclerosis, indicating that an abnormal immune response or thrombotic tendency caused by the vaccine was associated with the onset of symptoms in the right eye. Nevertheless, it cannot be ruled out that papillitis preceded the development of the present conditions and induced vascular occlusion due to increased pressure in the optic nerve.

Although all three vaccine preparations in the present case were from Moderna, there have been reports of adverse reactions to vaccines from other manufacturers, and it is possible that the same type of retinal arteriovenous occlusion may have developed in those cases. Our findings are similar to those of a 72-year-old man who developed arteriovenous occlusion with exudative retinal detachment 10 days after vaccination against COVID-19 (8). Other cases have also been reported by Lee and Ikegami (6, 9) and systemic thrombotic side effects or simultaneous development of arteriovenous occlusive disease (10) following vaccination against COVID-19 have also been noted. Vaccine-related inflammation and immune response may cause thrombophilia and simultaneous arteriovenous occlusion of the retina. However, in the present case, concomitant findings related to papillitis of the optic nerve were noted. There have been reports of vaccine-induced inflammatory findings in the optic nerve, such as optic neuritis and ischemic optic neuropathy; thus, it is possible that retinal arteriovenous occlusion and papillitis co-occurred in this case. When examining patients with similar conditions, it is important to keep in mind that adverse retinal reactions may develop in combination.

## Limitation

This case report had several limitations. First, the sudden loss of vision was considered to be an adverse reaction to the vaccine received 1 day prior, although no examination of retinal vessels was performed prior to the vaccination. Furthermore, findings showing incomplete CRAO and papillitis, in addition to CRVO, complicate the possible pathophysiology of the present case, making it difficult to determine whether the primary cause was thrombosis of retinal vessels or inflammation. It cannot be ruled out that non-ischemic optic neuropathy existed as a precursor lesion and that severe edema of the optic disc caused circulatory disturbance of the retinal vessels. Nevertheless, it is considered important to present these findings as there have been few reports of such sudden loss of vision, along with severe hemorrhage and edema in the retina occurring soon after vaccination. Reports of similar cases would be helpful to further elucidate the pathogenic factors in the present patient.

## Data availability statement

The original contributions presented in the study are included in the article/supplementary material. Further inquiries can be directed to the corresponding authors.

## Ethics statement

The studies involving humans were approved by Institutional Review Committee of the Hirosaki University Graduate School of Medicine. The studies were conducted in accordance with the local legislation and institutional requirements. The participants provided their written informed consent to participate in this study. Written informed consent was obtained from the individual(s) for the publication of any potentially identifiable images or data included in this article.

## Author contributions

AF: Data curation, Writing – original draft. YS: Writing – review & editing. NN: Data curation, Writing – review & editing. NK: Data curation, Writing – review & editing. SK: Writing – review & editing. SH: Data curation, Writing – review & editing. SU: Writing – review & editing.
